# Signalling need for care: a neglected functional role of medical treatment

**DOI:** 10.1093/emph/eoad024

**Published:** 2023-08-14

**Authors:** Mícheál de Barra, Kawthar Hakimy, Marijn de Bruin

**Affiliations:** Centre for Culture and Evolution, Brunel University London, London, UK; Division of Psychology, Brunel University London, London, UK; Health Psychology Group, University of Aberdeen, Aberdeen, UK; Radboud University Medical Centre, Radboud Institute of Health Sciences, IQ Healthcare, Nijmegen, Netherlands

**Keywords:** signalling theory, medical overuse, sick role, social support, over treatment

## Abstract

**Background and Objectives:**

While the primary goals of medical treatment are typically to shorten illness or relieve symptoms, we explore the idea that an important additional goal for some patients is to communicate their needs. Drawing on *signalling theory*, we argue that undergoing treatments can help patients legitimize their illness and thereby enable access to crucial support during convalescence.

**Methods and Results:**

Four pre-registered within-subjects experiments (*n* = 874) show that participants are more inclined to provide care to people who undergo treatment, especially when that treatment is painful. Results show this incentivizes the use of antibiotic treatments for viral infections as well as drug treatments for mental illness. A cross-sectional study of 194 chronic pain patients shows that those who experience stigma and doubt over the legitimacy of their illness are more likely to accept aversive treatments. Furthermore, two experiments (*n* = 653) indicate that subtle manipulations of one’s sense of social support may increase willingness to accept treatment.

**Conclusions and Implications:**

These results indicate that people make decisions to provide care in part based on the presence or absence of treatment and furthermore that patients’ treatment decision-making is informed by the social consequences of their choices. Signalling theory may help explain the surprising longevity of some ineffective and costly medical procedures.

## INTRODUCTION

What motivates the use of medical treatments? Most patients probably seek to alleviate symptoms, shorten illness or reduce the risk of death. Here, we explore the idea that an additional goal for patients may be to signal need to other people. This signalling motive may help explain patient preferences for treatments with few biomedical virtues, a puzzling feature of historical medical practice and an important problem in contemporary medicine.

Signalling theory, developed in evolutionary biology and economics, explores the conditions in which a *signal* receiver can be confident that messages from a *sender* are honest [1–3]. Signalling theory is relevant in healthcare because sick people need rest and care during their convalescence and they must convincingly communicate this need to would-be caregivers. Since the effects of illness are often subtle, and since rest and care are desired by many healthy people too, would-be caregivers are often uncertain about who needs care and the legitimacy of any given request for care. We argue that the act of undergoing treatment can help communicate one’s need for care to others.

Care by family members, friends and others is often essential during convalescence and, in its absence, mortality rates are markedly higher [4, 5]. There is substantial evidence of this caregiving in small-scale societies [6] and suggestive evidence in Neanderthals [7]. Though beneficial to the recipient, this care is often costly to provide, with careers experiencing higher mortality risk and poorer health [8, 9]. Since the effects of illness on the body can be both subtle and variable, would-be caregivers may not be able to ascertain who is sick and to what degree. Evidence of widespread uncertainty about the legitimacy of care requests can be observed in the testimonies of people who suffer from diseases that do not have visible manifestations—they often report that they find it difficult to gain emotional and practical support from others and their illnesses are perceived to lack legitimacy [10–13]. Doctors and medical researchers sometimes argue symptoms are a result of conscious or unconscious exaggeration [14, 15]. Whether or not these arguments are valid, they influence how healthcare workers and other members of the community perceive some illnesses. These findings suggest that potential caregivers are often sensitive to evidence about the legitimacy of illnesses. Medical treatment may provide this evidence.

Drawing on signalling theory [1], researchers [16, 17] have suggested that the act of undergoing medical treatment can provide credible evidence that someone has a legitimate illness. Individuals who are ‘genuinely’ sick should be more willing to undergo treatment than people engaging in illness deception. For sick people, investing time and energy into treatments that advertise their need is advantageous since these treatments enable access to potentially lifesaving care. Even risky or aversive treatments may be ‘good value’ if they enable the care patient’s need. For people engaging in illness deception, the benefits of care are typically smaller, and they may not justify this investment in treatment. Therefore, sick people should be more willing to endure the aversive treatment if it facilitates access to care [16]. Of course, if treatments are effective but have side effects, the marginal return on investing resources on treatment are even greater for sick people than for non-sick people since only they experience the curative effects. A toxic antibiotic might benefit someone with an infection but harm someone without one. But even in situations where the treatments are broadly ineffective or harmful, the critical importance of convalescent care means that sick people benefit from treatment acceptance and, the care it enables, in ways that others will not.

A key prediction of the signalling model of medicine is that people will be more inclined to provide resources to people who undergo treatment, especially aversive treatment. From the caregiver’s perspective, the fact that someone is willing to undergo treatment carries information value about that person’s health status and their need for care. This prediction is tested in Study 1. In Study 2, we examine the incentives that operate in two important areas of contemporary health care: the underuse of non-medical interventions for mental health problems and the overuse of antibiotics.

Cues differ from signals in that only the latter are produced *in order to* influence another individual. This attempt to influence need not be conscious or deliberate. Undoubtedly, medical treatments act as cues: our judgements about the severity of a friend’s back pain are likely to change if we learn they are due to undergo surgery. But the surgery only counts as *a signal* if our friend’s decision to undergo surgery is, in part, influenced by how the operation will change other people’s judgements of his pain. Study 3 explores this cue/signal distinction. In a sample of chronic pain patients, we test if patients whose friends, family and colleagues have withdrawn support or who directly suspect illness deception are more willing to use aversive treatments than patients whose network has fewer concerns about the legitimacy of their requests for support. In experimental studies, we explore how subtle manipulations of perceptions of social support influence willingness to undergo treatment.

All our studies’ hypotheses and analyses plans have been pre-registered and every pre-registered study we have conducted on this topic is described here, see https://osf.io/gs6wv/ for preregistrations, datasets and R analysis files.

## Studies 1a and 1b: Medicine as a cue to caregivers

Study 1a assesses if participants are more inclined to provide care to people who undergo (aversive) treatments. We additionally investigate if people are more likely to donate towards a charity related to a disease which was aversively treated compared to a disease that was not treated in the relevant vignette. Study 1b compares the effects of self-administered and doctor-prescribed treatments. A similar effect would indicate that the legitimizing force of treatments comes from patients’ willingness to endure the treatment rather than from doctors’ recommendations.

## METHODS

### Design

In this Study 1a, we used a repeated measures (fractional factorial) design. The first factor was disease, and there were three levels: back pain, acute respiratory infection (ARI) and stomach pain. The second factor was medical treatment, and there were also three levels: no treatment, treatment and aversive treatment. Participants saw each of the diseases and each of the medical treatment levels only once (i.e. they read three vignettes) and the allocation of medical treatment to disease was random. The three disease levels appeared in the same order in Study 1, but since the effects of disease on the dependent variables are not of theoretical interest, this confounding with order has little relevance. To assess if participants were aware of the study goal, we invited them to speculate on the purpose of the study in a free-text box after reading all three vignettes.

Study 1b differed from Study 1a in three ways. First, the vignettes were different and described a sick person who either underwent an aversive treatment recommended by a doctor, an aversive treatment they decided to undergo without a doctor’s recommendation or did not undergo treatment. The donation outcome measure was removed as it did not appear to be a sensitive measure of participants’ willingness to provide care.

### Participants

Participants were recruited from Prolific and completed the experiment for a reimbursement of 0.70. The only inclusion criterion was a minimum age of 25. This was intended to increase the likelihood that participants had workplace experience. This and subsequent studies were approved by the Brunel Research Ethics Committee.

### Vignettes

In Study 1a, the three vignettes described a colleague who claimed to be unable to work due to illness. Back pain, ARI and stomach pain were chosen for the vignettes since these illnesses are familiar, and, unlike a broken leg, for example, largely lack visible diagnostic symptoms. We created a version of the vignette in which the protagonist received no treatment, treatment or an aversive treatment. Prior work [16] suggests that treatments will have a more substantial influence on caregiving when there is ambiguity over the authenticity or severity of the illness. We created this ambiguity by, as mentioned earlier, choosing illnesses without apparent causes. We also included some contextual cues such as general work dissatisfaction or a tedious task to create the perception that illness deception is possible.

One possible confound is that vignette characters with harmful treatments are experiencing more ill health than patients with benign treatments, and therefore, may be more deserving of care, independent of any signalling effects. To mitigate this confound, we took the following steps: in the back pain vignette, we made it clear that the treatment only caused a temporary increase in suffering the previous day and should, therefore, be irrelevant to current decision-making. Similarly in the stomach pain condition, the aversive component—an endoscopy—was a brief procedure executed before the relevant caregiving opportunity. In the ARI vignette, the treatment’s aversive side effects—stomach pain and nausea—were included as additional symptoms of the disease in the no-treatment and treatment conditions. The vignette character, therefore, had the same number of health problems in all three conditions; see Table 1.

**Table 1.** Vignette stimuli used in Study 1a

Condition		Vignette
Back pain		
	No treatment	Your co-worker keeps complaining about back pain lately. The pain does not have any obvious cause and he seems to be able to walk without difficulty. Although it is an important week at work, he has been late to work every day due to the back problem. You keep having to stay till 9 pm at night to finish the work that he is not doing. Today, you overhear that your co-worker went to the doctor and *no medication was prescribed.*
	Treatment	As above, but last clause reads:*...they were prescribed strong prescription painkillers to take every night before bed.*
	Aversive treatment	As above, but last clause reads:*...they were prescribed strong prescription painkillers to take every night before bed. An unpleasant side-effect of the medicine is that he feels very nauseous and dizzy for a couple of hours after taking them.*
Acute respiratory illness		
	No treatment	Someone that you work with came into work this week sniffling a little. **She also has stomach pains and nausea**. For various reasons your colleague keeps missing work since she was hired two months ago. It seems she does not enjoy her job very much. And now this week you see she is leaving work early and missing important meetings. Today she has emailed in sick from work and attached a *sick note* from the local doctor. The doctor wrote that her problem was not very serious *and that he did not prescribe any antibiotics or medication.* Once again, you and your colleagues are going to have to work on Saturday to complete her tasks.
	Treatment	As above but italicised clause reads: …*and that he prescribed a course of strong antibiotics to take for her illness.*
	Aversive treatment	As above but the bold sentence was removed, and italicised clause reads:*...and that he prescribed a course of strong antibiotics to take for her illness. The doctor mentioned that these antibiotics are effective but will cause her stomach pains and nausea as a side effect.*
Stomach pain		
	No treatment	Your co-worker was due to give an important presentation to a group of clients. None of your team like giving these presentations, but it was his turn. He was not looking forward to it. A day before the presentation, your co-worker has come into work complaining of stomach pain. The next day, he calls in sick. You will need to take over the presentation. Your co-worker has a *sick note* from the doctor for one day’s sick leave. *They were examined by the doctor and no medication was recommended.*
	Treatment	As above but italicised sentence reads: *They were examined by the doctor and prescribed medication which is helping with their stomach pain.*
	Aversive treatment	As above but italicised sentence reads: *They were examined by the doctor with an endoscope (a camera tube that is pushed down the throat and into the stomach) and prescribed medication which is helping with their stomach pain.*

The text the participants saw did not include any italics or bold.

In Study 1b, the protagonist had either (a) no treatment, (b) treatments based on a doctor’s recommendation or (c) self-administered treatment, see Table 2. In the no-treatment condition, the protagonist’s symptoms were described and participants were informed that ‘she/he has a doctor’s appointment next week’. Treatment was not mentioned. In the self-treatment condition, the participants read that the protagonist had an appointment the next week but had begun a self-treatment that was both aversive and feasible to self-administer. The doctor-treated condition was identical except that the same treatment was described as a recommendation from the doctor.

**Table 2.** Vignette stimuli used in Study 1b

Condition		Vignette
Back pain		
	No treatment	Your co-worker keeps complaining about back pain which keeps him awake at night. He seems to be able to walk without any sign of a problem. Although it is a stressful week at work, he has been late every day due to the back problem. You keep having to stay till 9 pm at night to finish his work. *Today, you overhear that he has a doctor’s appointment next week.*
	Self-administered treatment	As above, but last sentence reads: *Today, you overhear that he has a doctor’s appointment next week. Till then, he has started sleeping on a hard wooden floor rather than on a mattress to help him sleep.*
	Doctor-administered treatment	As above, but last clause reads: *Today, you overhear that following his doctor’s advice, he has started sleeping on a hard wooden floor rather than on a mattress to help him sleep.*
Irritable bowel syndrome		
	No treatment	Your co-worker was due to give an important presentation to a group of clients. None of your team like giving these presentations, but it was his turn. Your co-worker recently mentioned he gets abdominal cramps and bloating. Now, on the day of his presentation, he calls in sick due to these symptoms. You will need to take over the presentation. *He has a doctor’s appointment next week.*
	Self-administered treatment	As above, but the last sentence reads: *He has a doctor’s appointment next week. He has given up eating all wheat (bread, pasta and biscuits) to try to alleviate the problem.*
	Doctor-administered treatment	As above, but the last clause reads: *Following his doctor’s recommendation, he has given up eating all wheat (bread, pasta and biscuits) to try to alleviate the problem.*
Injury		
	No treatment	For various reasons your colleague keeps missing work since she was hired 2 months ago. She does not seem to like her job. This week, she has a shoulder injury and has been leaving work early. Once again, you and your colleagues are going to have to work on Saturday to complete her tasks. *She has a doctor’s appointment next week.*
	Self-administered treatment	As above, but last sentence reads: *She has been taking 10-minute baths in freezing iced water every morning to quicken her recovery and has a doctor’s appointment next week.*
	Doctor-administered treatment	As above, but last sentence reads: *Following her doctor’s recommendation, she has been taking 10-minute baths in freezing iced water every morning to quicken her recovery.*

The text the participants saw did not include any italics.

To encourage participants to pay attention to the vignettes, we asked them to ‘describe what happened to your co-worker in the previous extract’ in a free text box after reading the vignettes and completing the outcome variables. The responses were not analyzed.

The Psytoolkit [18, 19] syntax for recreating this and all subsequent experiments is available on the Open Science Framework [https://osf.io/gs6wv/].

### Outcome variables

The caregiving index was the primary outcome variable and consisted of seven questions, each accompanied by a 5-point Likert scale (strongly agree, agree, neither agree nor disagree, disagree, strongly disagree). Example items include: *This co-worker is definitely ill*; *My team and I should take over all their work-related responsibilities until they recover fully*; and *It was fully acceptable for them to be late/absent*. The full list of items is available in Supplementary File, Section A. Item order was randomized between participants and between vignettes. Cronbach’s α s of > 0.89 indicated good internal consistency across the seven items. Given that this is a new measure of care, we examined the convergent and divergent validity of the caregiving index on a separate sample. The methods and results are presented in detail in the Supplementary File, Section B. In brief, scores on the caregiving index correlate (*r* = 0.36) with *principle of care*, an index of the position that one is morally obliged to help those in need. It is also associated with high scores on the empathic concern sub-scale of the interpersonal reactivity index and lower scores on the distrust of others sub-scale of the Machiavellianism scale. These latter two associations are not statistically significant.

The second outcome variable consisted of a single question at the end of the study, inviting participants to vote for one of three charities. The charity with the most votes received a donation of £25. The charities had a health focus that linked them to the three clinical areas of the vignettes: back pain [described as ‘A charity that funds research on back pain (Arthritis Research UK)’]; ARI (‘a charity that funds research on colds, flu and other respiratory diseases (British Lung Foundation)’)] and stomach pain (finally ‘a charity that funds research on stomach/digestive problems (Core—Fighting Digestive Diseases))’. Hence, this item is a behavioural outcome measure of willingness to transfer resources to charities linked to the problems experienced by the vignette characters.

### Analysis

A linear mixed effects model in which scores were nested within participants was used to test the effects of the disease manipulation on the caregiving index scores. Participants are modelled as random effects while manipulation (no treatment vs treatment vs aversive treatment), disease and sex are modelled as fixed effects. The fit of a full model (${\hat{care}}_{i}=manipulation_{i}+disease_{i}+sex_{i}+participant_{j\left[i\right]}$) was compared with a model without the manipulation (${\hat{care}}_{i}=disease_{i}+sex_{i}+participant_{j\left[i\right]}$) using an AIC, BIC and likelihood ratios. Superior fit of the full model would indicate that the manipulation influences scores on the care index. Post-hoc tests were used to test that the differences are in the predicted direction.

Multinomial logistic regressions were used to assess if the probability of donating to a charity X was higher after reading a vignette featuring someone who experiences disease X plus treatment or aversive treatment versus after reading a vignette featuring someone who experiences disease X without treatment. We compare the fit of a model with the key predictor variable, presence/absence of treatment, to a model without this predictor. Since each participant makes a single donation decision, this is a between-subjects analysis.

There were no modifications to the pre-registered analysis plan.

## RESULTS

In total, 248 peoples (63% women) completed the study and the mean age was 37.04 (SD = 10.63). While we did not collect participant nationality, the population from which they are drawn, Prolific.ac.uk participant pool, are mostly resident in the UK (31%) or the US (28%) [20].

In both the experiments, a full model including a fixed effect for manipulation provided a better fit to the data than a comparison model without this manipulation variable (Study 1a: likelihood ratio = 137.56, *P* < 0.0001, full and comparison model AIC: 1692, 1558.4, full and comparison model BIC: 1719.3, 1594.9, Study 1b: likelihood ratio = 46.71, *P* < 0.0001, full and comparison model AIC: 1235.1, 1192.4, full and comparison model BIC: 1261.6, 1227.7), see Table 3.

**Table 3.** Full and comparison linear mixed effects models predicting care for untreated, benign treatment and aversive treated targets, see Study 1a

	Full model			Model without treatment		
Coefficient	Estimates	Conf. Int (95%)	*P*	Estimates	Conf. Int (95%)	*P*
Intercept	2.18	2.04–2.31	**<0.001**	2.56	2.44–2.68	**<0.001**
Treatment versus no treatment	0.44	0.32–0.55	**<0.001**			
Aversive treatment versus no treatment	0.72	0.60–0.83	**<0.001**			
Back pain versus ARI	0.66	0.55–0.77	**<0.001**	0.63	0.50–0.76	**<0.001**
Stomach pain versus ARI	0.23	0.11–0.34	**<0.001**	0.26	0.13–0.39	**<0.001**
Men versus women	0.02	−0.13 to 0.17	0.818	0.02	−0.13 to 0.17	0.786
Random effects						
σ^2^	0.38			0.51		
τ_00_	0.20 _ID_			0.16 _ID_		
ICC	0.34			0.24		
N	243 _ID_			243 _ID_		
Observations	706			706		
Marginal R^2^/Conditional R^2^	0.211/0.483			0.093/0.310		

*P* values less than .05 are in bold.

**Figure 1.**
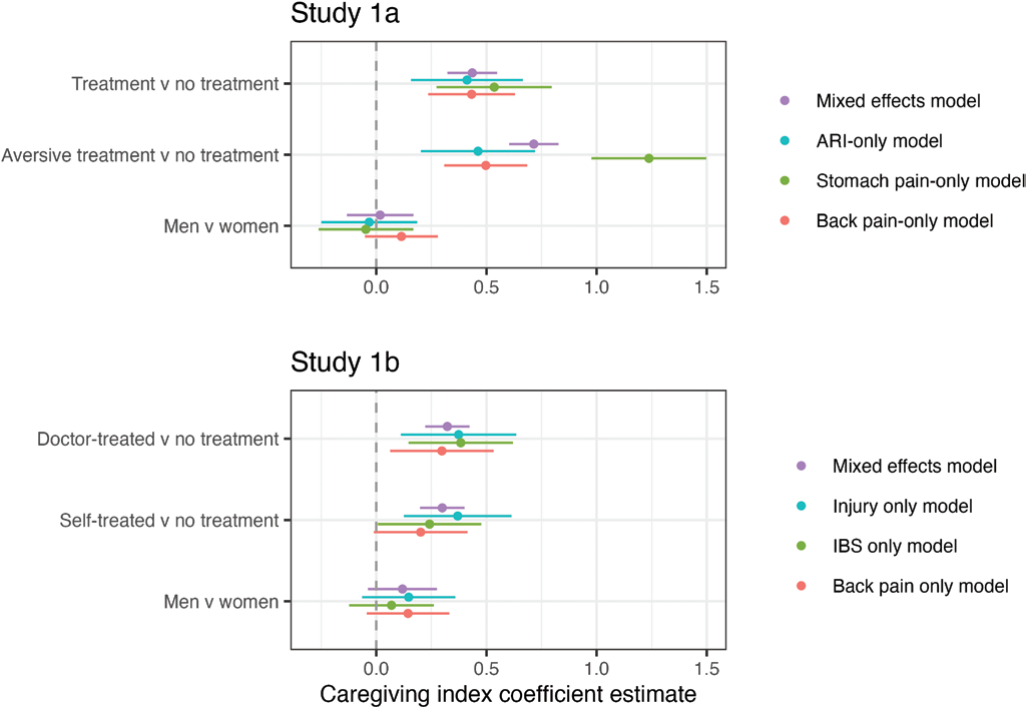
Estimating caregiving index scores after aversive and self-administered treatment. Note: Standard errors shown with lines. Green, blue and red lines show results of regression models predicting responses to one disease vignette only

In Study 1a, post-hoc tests indicated a significant difference in the predicted direction between treatment and no treatment (coefficient difference = 0.44, *P* < 0.0001) and between treatment and aversive treatment (coefficient difference = 0.28, *P* < 0.0001). The purple points/lines in Fig. 1 show the fixed effect coefficient estimates from the multilevel model. Caregiving scores for aversive treatments were 0.72 points higher than for no treatment vignettes. For reference, the standard deviation of caregiving index scores is 0.8.

In Study 1b, there was a statistically significant difference between self-treatment and no treatment (coefficient difference = 0.32, *P* < 0.0001) and between prescribed-treatment and no treatment (coefficient difference = 0.30, *P* < 0.0001), see Table 4. There was no statistically significant difference between self-treatment and prescribed treatment (coefficient difference = −0.02, *P* = 0.89).

**Table 4.** Full and comparison linear mixed effects models predicting care for doctor-treated, self-treated and untreated targets, see Study 1b

	Full model			Model without treatment		
Coefficient	Estimates	Conf. Int (95%)	*P*	Estimates	Conf. Int (95%)	*P*
Intercept	2.71	2.58–2.84	**<0.001**	2.91	2.79–3.03	**<0.001**
Doctor-treated versus no treatment	0.32	0.22–0.42	**<0.001**			
Self-treated versus no treatment	0.30	0.20–0.40	**<0.001**			
Back pain versus injury	0.09	−0.02 to 0.19	0.096	0.08	−0.02 to 0.19	0.128
IBS versus injury	0.26	0.16–0.36	**<0.001**	0.28	0.17–0.38	**<0.001**
Men versus women	0.12	−0.04 to 0.28	0.137	0.12	−0.04 to 0.28	0.132
Random effects						
σ^2^	0.27			0.30		
τ_00_	0.23 _ID_			0.21 _ID_		
ICC	0.46			0.42		
N	207 _ID_			207 _ID_		
Observations	611			611		
Marginal R^2^/Conditional R^2^	0.072/0.499			0.032/0.436		

*P* values less than .05 are in bold.

Regarding the donations to charity in Study 1a, the full model including a fixed effect for manipulation did not provide a better fit to the data than a model without the manipulation variable (likelihood ratio = 0.21, P=0.99, full and comparison model AIC: 493.12, 485.33). Thus, the presence of treatment has no effect on participants’ propensity to vote to donate £25 to a charity related to the treated disease.

To assess if participants had inferred the experimenter’s intentions, and thereby might have biased the results, we asked participants ‘What question do you think the researchers are trying to answer?’ at the end of Study 1a and provided a free text box. Nine participants (4% of total) correctly guessed that the aim was to assess caregiving when treatment levels varied. Excluding those participants from the analyses did not affect our results.

## DISCUSSION

Participants were more inclined to provide care to people after medical treatments, especially after aversive treatments. The aversiveness of the treatment appeared most important in the stomach pain vignette; perhaps the treatment was more viscerally unpleasant or the suggestion of illness-deception was stronger in this particular vignette. No effect of the manipulation of participant donations to charity was observed. In hindsight, reading short vignettes about a specific person with symptoms may be too weak a manipulation to influence general attitudes towards charities loosely associated with these symptoms.

### Studies 2a and 2b: Applications of signalling to mental health care and antibiotic stewardship

Study 2 examines how signalling concerns might incentivise suboptimal treatment choice in two important health domains: drug treatment of mental health and antibiotic treatment of viral infections.

Many health problems, and particularly mental health problems, can be treated with drugs or with psychological treatments such as therapy or exercise. These treatments might have different capacities to signal need: psychological treatments such as therapy, exercise or self-administered cognitive behavioural therapy (three treatments recommended by the NHS) are unlikely to appear harmful and may not be effective at communicating need. In contrast, psychiatric drugs are perceived to be dangerous [21, 22]. In Study 2a, we test the hypothesis that participants will be more likely to care for people who undergo pharmacological treatments than to people who undergo psychological treatments or no treatment.

Antibiotic resistance represents an important threat to human longevity and well-being [23, 24]. In Study 2b, we examine if the ability of medical treatments to signal need incentivizes excessive antibiotic use. Two important antibiotic stewardship measures are (i) raising the threshold for prescriptions so that they are less often used for mild bacterial or viral infections and (ii) delaying antibiotic use for a period to allow self-limiting illness to heal without intervention. Post-dated prescriptions are one way to delay use (see e.g. [25]). Non-use and delayed-use may provide patients with a weaker signal of need because they could be seen to indicate a minor infection. Antibiotic use, on the contrary, may suggest a health problem that justifies the population-level costs as well as the individual-level harms. Hence, antibiotic use may be a more effective signal of the need for care than non-use or delayed use.

## METHODS

Studies 2a and 2b were identical to Study 1b, except for the vignette content. In Study 2a, participants read three vignettes about characters with mental health problems who underwent either a drug treatment, a psychological treatment or no treatment. After each vignette, participants completed the caregiving index. Both the symptoms profile and the treatment regimens were based on descriptions from the NHS website, see Table 5.

**Table 5.** Vignette stimuli used in Study 2a

Condition		Vignette
Anxiety		
	No treatment	Your colleague keeps missing deadlines and does not seem to enjoy her job very much. This week, she has been complaining of feeling anxious and being unable to sleep. She has been leaving work earlier than usual while you and your colleagues have had to stay later than usual each day to do her work. *You hear that she had been to the doctor.* You will have to finish her projects again.
	Non-pharmacological treatment	As above, but italicized sentence reads: *You hear that she had been to the doctor who recommended a self-help book.*
	Pharmacological treatment	As above, but italicized sentence reads: *You hear that she had been to the doctor who recommended diazepam, a prescription drug.*
Depression		
	No treatment	Your colleague has become irritable and snaps at people when spoken to. He also comes late and always seems to be upset and down all the time. Worst of all, he has missed several deadlines and you and your team have been working overtime to make up for his failings. *Today you overhear that he has been to see a doctor about his problems.*
	Non-pharmacological treatment	As above, but final sentence reads: *Today you overhear that he has been to see a doctor who recommended a therapist who he now sees.*
	Pharmacological treatment	As above, but final sentence reads: *Today you overhear that he has been to see a doctor who has prescribed him a course of antidepressant medication to help with his condition.*
Chronic fatigue syndrome		
	No treatment	Your co-worker was due to give an important presentation to a group of clients. None of your team like giving these presentations, but it was her turn. She was not looking forward to it. A day before the presentation, your co-worker has again come into work complaining of tiredness and headaches. The next day, she calls in sick. You will need to take over the presentation. You find out that she has been to the doctor.
	Non-pharmacological treatment	As above, includes an additional sentence: *Following his recommendation, she has been doing some light exercise to help with her problem.*
	Pharmacological treatment	As above, includes an additional sentence: *Following his recommendation, she has been prescribed medication to help with her problem.*

The text the participants saw did not include any italics.

In Study 2b, the vignette character either received antibiotics immediately, received a delayed prescription for antibiotics, or did not receive antibiotics, see Table 6. We sought to include descriptions of treatments in the vignettes that closely mirror what might happen in clinical contexts. We therefore based the treatments on clinical trials assessing these stewardship measures. The treatment elements of the cough vignettes were based on the descriptions of the interventions in three arms of a trial by [26]. In this trial, participants were allocated to conditions including no offer of antibiotics, a delayed offer of antibiotics or an immediate offer of antibiotics. In the urinary tract infection vignettes, the treatments were based on a trial by [27] in which patients in a delayed condition were offered antibiotics in a few days if they did not improve or were provided immediate antibiotics. We also included a no-antibiotic treatment in which, consistent with the UK’s NICE guidelines, patients were ‘advised that paracetamol can be used for pain relief’. Thus, all the three arms are plausible healthcare experiences for people in the UK with UTIs. The final set of treatments was derived from an RCT on management strategies for sore throat [28] in which patients were offered immediate antibiotics, antibiotics if symptoms failed to settle in three days or no antibiotics. Similar interventions have been tested in other trials [25].

**Table 6.** Vignette stimuli used in Study 2b

Condition		Vignette
Cough		
	No antibiotics	Your co-worker was due to give an important presentation to a group of clients. None of your team like giving these presentations, but it was his turn. Now, on the day of his presentation, he emails to say he has a cough and cannot give the presentation. *He went to the doctor who did not prescribe anything.* You will need to take over the presentation.
	Delayed antibiotics	As above, but italicized text reads: *He went to the doctor who told him that he should come back for antibiotics in a few days if he felt substantially worse.*
	Antibiotics	As above, but italicized text reads: *He went to the doctor who prescribed a course of antibiotics.*
Urinary tract infection		
	No antibiotics	For various reasons your colleague keeps missing work since she was hired two months ago. She does not seem to like her job. This week, she says she has a urinary tract infection and has asked to leave work early. Once again, you and your colleagues are going to have to work on Saturday to complete her tasks. She has been to a doctor who advised *that paracetamol can be used for pain relief.*
	Delayed antibiotics	As above, but italicized text reads: *…that paracetamol can be used for pain relief and said he would prescribe antibiotics in a couple of days if she did not improve.*
	Antibiotics	As above, but italicized text reads: *…that paracetamol can be used for pain relief and prescribed a course of antibiotics.*
Throat infection		
	No antibiotics	Your co-worker keeps complaining about a sore throat which keeps him awake at night. He seems to be able to talk without any sign of a problem. Although it is a stressful week at work, he has been late every day. You keep having to stay till 9pm at night to finish his work. The doctor who saw him *did not recommend antibiotics.*
	Delayed antibiotics	As above, but the italicized text reads: …*recommended that he collect antibiotics from the surgery if symptoms were not starting to settle within three days.*
	Antibiotics	As above, but italicized text reads: …*recommended antibiotics.*

The text the participants saw did not include any italics.

## RESULTS

In Study 2a, 204 participants had a mean age of 35.05 (SD = 9.06). There were 115 (56%) female participants. In Study 2b, 316 participants had a mean age of 38.19 years (SD = 11.85). Gender was reported as male by 126 participants and as female by 190 participants.

In both studies, the null models had a poorer fit than the models including manipulation (Study 2a: likelihood ratio = 43.78, P<0.0001, full and comparison model AIC: 1313.8, 1274, full and comparison model BIC: 1340.2, 1309.2, Study 2b: likelihood ratio = 82.77, *P* < 0.0001, full and comparison model AIC: 1530.3, 1451.6, full and comparison model BIC: 1556.9, 1486.9). See Tables 7 and 8 for details.

**Table 7.** Full and comparison linear mixed effects models predicting care for targets with mental health difficulties and pharmacological, non-pharmacological or no treatment, see Study 2a

	Full model			Model without treatment		
Coefficient	Estimates	Conf. Int (95%)	*P*	Estimates	Conf. Int (95%)	*P*
Intercept	3.12	2.98–3.26	**<0.001**	3.19	3.07–3.32	**<0.001**
Non-pharm versus no treatment	−0.02	−0.14 to 0.09	0.703			
Pharma versus no treatment	0.33	0.21–0.44	**<0.001**			
CFS versus anxiety	−0.06	−0.18 to 0.05	0.282	−0.03	−0.15 to 0.09	0.663
Depression versus anxiety	0.17	0.06–0.29	**0.003**	0.22	0.10–0.34	**<0.001**
Men versus women	−0.12	−0.28 to 0.04	0.151	−0.12	−0.27 to 0.04	0.157
Random effects						
σ^2^	0.33			0.37		
τ_00_	0.22 _ID_			0.20 _ID_		
ICC	0.39			0.36		
N	203 _ID_			203 _ID_		
Observations	603			603		
Marginal R^2^/Conditional R^2^	0.069/0.436			0.026/0.374		

*P* values less than .05 are in bold.

**Table 8.** Full and comparison linear mixed effects models predicting care for targets with different kinds of antibiotic care, see Study 2b

	Full model			Model without treatment		
Coefficient	Estimates	Conf. Int (95%)	*P*	Estimates	Conf. Int (95%)	*P*
Intercept	3.14	2.99–3.29	**<0.001**	2.87	2.74–3.00	**<0.001**
Delayed antibiotics versus antibiotics	−0.12	−0.26 to 0.01	0.077			
No antibiotics versus antibiotics	−0.64	−0.77 to −0.50	**<0.001**			
Throat versus cough	−0.01	−0.14 to 0.13	0.940	0.01	−0.14 to 0.17	0.852
UTI versus cough	0.09	−0.05 to 0.22	0.225	0.12	−0.04 to 0.27	0.135
Men versus women	−0.02	−0.17 to 0.13	0.797	−0.03	−0.18 to 0.12	0.717
Random effects						
σ^2^	0.51			0.62		
τ_00_	0.13 _ID_			0.09 _ID_		
ICC	0.21			0.13		
N	211 _ID_			211 _ID_		
Observations	614			614		
Marginal R^2^/Conditional R^2^	0.109/0.292			0.004/0.133		

*P* values less than .05 are in bold.

**Figure 2.**
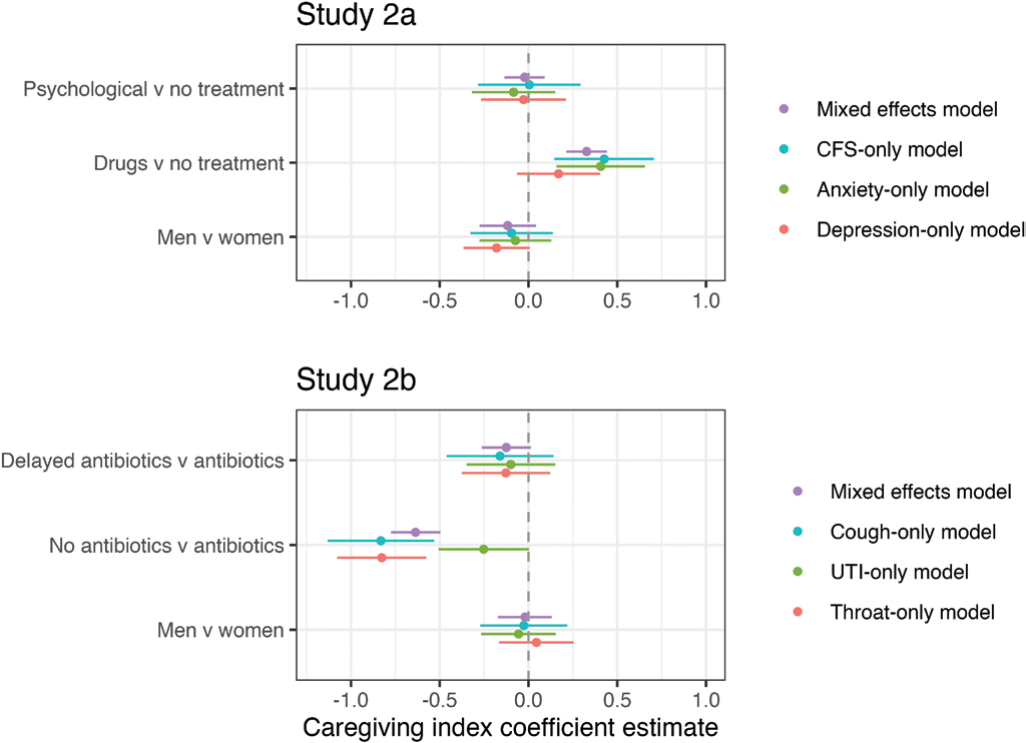
Estimating caregiving index scores: mental health and antibiotic use. Note: Standard errors shown with lines. Green, blue and red show results of regression models predicting responses to one disease vignette only

As Fig. 2 illustrates psychological treatments had little effect on caregiving scores (coefficient difference = −0.02, P=0.92). Drug treatments, on the contrary, increased caregiving scores relative to no treatment (coefficient difference = 0.33, *P* < 0.0001) and to psychological treatment (coefficient difference = 0.35, P<0.0001).

The results of Study 2b indicate that the absence of antibiotics caused a.64 reduction in caregiving relative to immediate antibiotics. A post-hoc test also indicated the difference between delayed and no antibiotics was statistically significant (coefficient difference = −0.51, *P* < 0.001.). There was no statistically significant difference between immediate antibiotics and delayed antibiotics. A sensitivity analysis with 300 Monte Carlo simulations using the SIMR package in R [29] indicated that this study had 80% power to detect changes of 0.18 units on the 1 to 5 caregiving scale. Therefore, a difference between immediate and delayed prescriptions that is smaller than 0.18 units may well be missed by these analyses.

## DISCUSSION

Results of Study 2a indicate that the participants were more likely to provide care when the vignette protagonist underwent a drug treatment. There was no evidence that psychological treatments had signalling value. This suggests that some patients experience an incentive to reject psychological treatments and accept pharmacological treatments.

Participants perceived that an illness was more legitimate when the sick person received immediate or delayed antibiotics, supporting the hypothesis that antibiotic treatments have signalling value. If this finding generalises to real-world settings, antibiotic stewardship measures may benefit from considering how to provide people with evidence of need without antibiotics. The results are consistent with research indicating that ‘legitimation’ of illness to employers and others is an important reason for seeking treatment [28].

## Studies 3a, 3b and 3c: Stigma, illness legitimacy and patient preferences for aversive treatments

Studies 1 and 2 show that people are more willing to provide care to those who undergo a medical treatment, creating an incentive for over-treatment. We next focus on how people respond to these incentives. In Study 3a, an observational study of people with chronic pain, we examine if patients are more inclined to accept aversive treatments if their social contacts suspect illness deception or are reacting negatively to their illness. Specifically, we assess if participants who report that others do not see their illness as legitimate, or who experience illness stigma, will be more accepting of treatments, and in particular aversive treatments, than participants whose friends and family feel their illness is legitimate.

Then, in two experimental Studies 3b and 3c, we randomise chronic pain patients to complete an illness-legitimacy measure or a control chronotype measure and then assess their willingness to accept treatments. We hypothesise that spending a few moments reflecting on the perceived legitimacy of one’s illness will result in an increase in willingness to accept medical treatment.

## METHODS

### Sample

In all studies, recruitment was limited to people who reported 3 months or more of pain on a prolific.ac pre-screening questionnaire.

### Measures

In all three studies, treatment acceptance was assessed by asking participants if they would accept a series of treatments in order to access one additional pain-free day during their worst period of pain. Four items assessed preferences for benign treatments with low-signalling value (e.g. ‘I would do 1 hour of intense exercise every single day’ or ‘I would download and use a pain-related smartphone app for 30 minutes every day’) and the other four items assess preferences for more aversive treatments which presumably have greater signalling value (e.g. ‘I would undergo a minor surgery which will result in some visible scarring’ or ‘I would take a drug that carries a risk of stroke’). See Part C of the Supplementary Materials for the full list of items and the psychometric properties of the scales. The item order presentation was randomized between participants.

To assess chronic pain levels, use used the Chronic Pain Grade Scale, in its original (Studies 3a or 3b [30],) or newly revised form (Study 3c [31]). We also asked participants to describe the nature, duration, and location of their pain in free-text boxes.

In the correlational study, we included a measure of stigma, the eight-item Stigma Scale for Chronic Illnesses (SSCI-8 [32]) to assess people’s sense that their value as a social partner has declined due to their illness. The SSCI-8 assess a sense that other people are avoiding or rejecting you (e.g. ‘Because of my pain/illness, I felt left out of things’ or ‘Because of my pain/illness, people were unkind to me’) and negative emotional responses to these loss of social value (‘I felt embarrassed about my pain/illness’).

To more directly measure the perception that other people think one’s illness is legitimate, the correlational Study 3a used a 6-item measure derived from the caregiving index used in Studies 1 and 2. Items included ‘Other people sometimes think I am lazy rather than sick’, ‘If people took my pain/illness more seriously, I would have greater support’ and ‘Some friends and family members are sceptical of my pain/illness’.

The experimental Studies 3b and 3c used the same legitimacy measure as an experimental manipulation. In these between-subjects studies, participants were randomized to complete the legitimacy index or a control chronotype index with the same number of items. Since the results of Study 3b were somewhat ambiguous, we repeated the study and included slight changes to the items and a free text box in which participants were invited to explain their responses. This was intended to increase the duration and intensity of the manipulation.

To summarise, Study 3a asks if low-legitimacy and stigma predict higher levels of treatment acceptance in a correlational study. In Studies 3b and 3c, we examine if a manipulation to legitimacy (i.e. completing the legitimacy index) increases people’s tendency to accept treatments.

### Results

In total, 835 participants were recruited for Study 3. This excludes 64 participants who had scores 1 (‘low disability low intensity pain’) on the Chronic Pain Grade Scale in studies or 0 (‘chronic pain absent’ on the revised measure). Participant’s mean age was 33.94 (SD = 11.04) (SD) and 54.65% were women. In total, 65.27% experienced musculoskeletal chronic pain (e.g. back pain, joint pain, arthritis, fibromyalgia), 13.48% reported pain the internal organs (e.g., stomach pain, prolonged period pain) and 14.92% experienced pain the head/mouth/ears.

**Figure 3.**
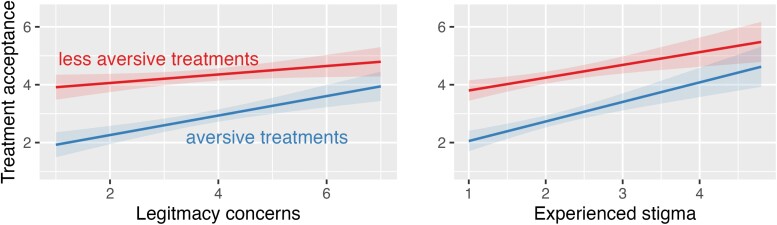
Interaction plots showing legitimacy concerns influence aversive treatment concerns more than less aversive treatments

The cross-sectional data from Study 3a indicate that 182 people with chronic pain are more inclined to accept treatment if they report experiencing illness stigma or if they concern about the perceived legitimacy of their illness, see Table 9. The effect of low legitimacy appears more pronounced for on aversive treatments, see Fig. 3. (Note this analysis provides a clearer test of our hypotheses than the preregistered plan, but the qualitative patterns are similar, see Supplementary File, Part D).

**Table 9.** What predicts treatment acceptance among 161 people with chronic pain?

Coefficient	Estimates	Estimates	Estimates	Estimates
Intercept	3.42 ^***^ (2.94–3.89)	3.77 ^***^ (3.21–4.33)	3.11 ^***^ (2.62–3.59)	3.36 ^***^ (2.78–3.94)
Treatment is aversive	−1.48 ^***^ (−1.72 to −1.23)	−2.18 ^***^ (−2.82 to −1.54)	−1.48 ^***^ (−1.72 to −1.23)	−1.98 ^***^ (−2.66 to −1.30)
Low legitimacy	0.24 ^***^ (0.13–0.36)	0.15 ^*^ (0.01–0.29)		
L. legitmacy*aversivness		0.19 ^*^ (0.03–0.35)		
High stigma			0.56 ^***^ (0.36–0.76)	0.44 ^***^ (0.19–0.69)
H. stigma*aversivness				0.23 (−0.06 to 0.53)
Random effects				
σ^2^	1.39	1.36	1.39	1.38
τ_00_	0.68 _id_	0.70 _id_	0.60 _id_	0.61 _id_
ICC	0.33	0.34	0.30	0.31
N	182 _id_	182 _id_	182 _id_	182 _id_
Observations	364	364	364	364
Marginal R^2^/Conditional R^2^	0.246/0.494	0.253/0.506	0.275/0.494	0.278/0.498

*P < 0.05, **P < 0.01 and ***P < 0.001.

Randomising chronic pain participants to complete an illness legitimacy measure (intended to briefly foreground people’s legitimacy concerns) appeared to increase people’s willingness to accept aversion treatments. In Study 3b, the effect of this manipulation was in the predicted direction but not statistically significant. We therefore repeated the experiment (Study 3c) with minor modifications intended to increase the strength of the manipulation, resulting in larger effect sizes and statistically significant differences, see Table 10. Models including an interaction term did not indicate a stronger effect of the manipulation on more aversive therapies.

**Table 10.** Results of experiments 3b and 3c. Predicting chronic pain patients’ preference for treatments

	Study 3b		Study 3c	
Coefficient	Estimates	*P*	Estimates	*P*
Intercept	4.76 (4.56–4.97)	**<0.001**	4.45 (4.206–4.65)	**<0.001**
Randomised to complete legitimacy measure	0.19 (−0.07 to 0.45)	0.145	0.28 (0.03–0.54)	**0.030**
Treatment is aversive	−2.09 (−2.28 to −1.90)	**<0.001**	−1.95 (−2.10 to −1.79)	**<0.001**
Random effects				
σ^2^	1.43		1.08	
τ_00_	0.65 _id_		0.91 _id_	
ICC	0.31		0.46	
N	316 _id_		340 _id_	
Observations	632		680	
Marginal R^2^/Conditional R^2^	0.346/0.550		0.327/0.634	

*P* values less than .05 are in bold.

## Discussion

Results are consistent with the theory that aversive treatments are deployed by people who struggle to gain support from others as a signal of authenticity.

## General Discussion

These experiments show consistent support for the hypothesis that would-be caregivers use medical treatments—especially aversive treatments—as evidence of illness authenticity. A cross-sectional study and two experiments suggest that patient’s preferences may be shaped by these incentives: a perception that other people do not see your illness as legitimate is associated with greater preference for aversive treatments. This signalling incentive could lead people to consume more medical treatments than expected from a strictly biomedical perspective.

Strictly speaking, acceptance of a treatment provides information about the authenticity of someone’s *need for care*, but not necessarily about the authenticity about the specific cause of that need for care. People with somatization disorders, for example, often undergo procedures [33] which have little biomedical effect but that may have some positive influence on their social position and potentially their self-image. In some circumstances, even costly and harmful treatment may be justified for both sick and non-sick individuals. Shooting oneself in the hand may be a rational ‘medical’ intervention if the pay-off includes exemption from military service.

While we have stressed the importance of aversive treatments that deter all but those to stand to gain significantly from caregiving, there are likely be a broad range of conditions where low-cost signals are preferable. In some situations, the costs of providing support are so low that caregivers may be quite willing to help despite the risk of illness deception [16]. Similarly, if honesty has been demonstrated in other past interactions or if the caregivers anticipate reciprocity, low-cost but potentially ‘fakable’ communication may be sufficient to advertise a need for care. In these contexts, undergoing benign treatment (such as homoeopathy or over-the-counter medicines) may adequately communicate ones need. In the varied circumstances of inter-personal dependency that illness creates, communication of need will likely take many forms including low- and high-cost signals, and honest and dishonest signals [2, 3, 16]. Moreover, medical treatment is but one way in which people may endure bodily harm to demonstrate need: self-harm and suicidality have also been proposed as acts which enhance credibility and advertise need [34, 35].

Our results have implications for healthcare. Patients with poor-mental health often use pharmacological treatments over behavioural and psychological treatments for mental health problems, despite the fact that pharmacological treatments are sometimes less effective and more harmful [36], with side effects such as gastrointestinal problems, weight gain and sexual dysfunction [37]. Our results suggest an explanation for this pattern of behaviour: because they are perceived as harmful [21, 22], pharmacological treatments are more effective in signalling to others that one has a legitimate need for care. Exercise, on the contrary, may signal vitality and capacity rather than illness, reducing the perception of need. By better understanding the social consequences of treatment choice, researchers may be able to better understand how to develop non-pharmacological interventions that do not undermine user’s ability to recruit care from others.

Over 80% of human antibiotic use occurs in primary care [38], where unnecessary prescriptions for infections such as colds and cough are common. These results support our hypothesis that patients are incentivized by the signalling value of treatments to seek antibiotic treatment irrespective of their curative value. Consistent with this, educational interventions that successfully influence patients’ knowledge, attitudes and beliefs about antibiotics appear to have little effects on prescription rates [39], perhaps because they do not address the underlying motive for treatment.

More broadly, signalling concerns may be one important force driving demand for treatments that are, from a strictly biomedical perspective, unnecessary. Medical procedures with no or even negative direct effects are strikingly common across cultures and through history. Today, ‘low-value’ treatments are an important cause of rising health care costs [40]. The Institute for Medicine, for example, estimates that $210 billion is spent on biomedically unnecessary treatment each year in the US alone [41]. If we look beyond conventional medicine to traditions that make less use of robust tools for evaluating outcomes, the scale of overtreatment is larger still. Our results suggest that when signalling considerations are present, the marginal value of additional medical consumption is higher. We might expect individuals to undergo treatments to undergo treatments with limited or negative biomedical action if the treatment enables them to credible signal a need for care. Of course, treatments with biomedical as well as social benefits will be preferred, especially since these benefits are only going to be realised by the sick individuals. However, our read of contemporary as well as historical medicine is that effective treatments have been unavailable for many conditions [42] (though there are, of course, some effective treatments with deep roots in the human past, see e.g. [43] or [44]).

Signalling concerns may influence the kinds of treatments patients prefer as well as the quantity. All else equal, treatments perceived to be harmful and risky may have an advantage over ones perceived to have few risks or side effects. More observable treatments (splints, operations, etc.) present greater scope for displaying need than more subtle privately consumed medicines.

Study 3a usefully demonstrates the boundary conditions of the signalling model. Firstly, chronic pain patients with few concerns about legitimacy and who experience little stigma are less likely to seek aversive care. Secondly, signalling concerns are less likely to drive overuse of benign treatments such as homoeopathy. Instead, we expect signalling concerns to play an important role in patient preferences for extensive diagnostics (e.g. scans), surgery and other kinds of invasive and potentially harmful treatments.

Our study used pre-registered experimental methods to test predictions grounded in well-developed theories [1, 16] with real-world relevance. However, caution is warranted when generalizing online studies to health-seeking behaviour and caregiving beyond the lab. Another limitation of this study is that we used a novel self-reported outcome measure, the caregiving index, reflecting people’s willingness to provide care. However, our validation work suggests good internal consistency and reliability of the scale, as well as convergent validity.

## Conclusions

People appear to be more willing to provide care to people with ambiguous symptoms who undergo an aversive treatment. This, in turn, serves as an incentive for the use of medical treatments, independent of their effectiveness. Treatments can provide a badge of authenticity, enabling patients to persuasively communicate their need and enabling caregivers to allocate their resources efficiently. This may be an important but overlooked mechanism that can explain some patients’ treatment preferences.

## Supplementary Material

eoad024_suppl_Supplementary_File
