# Supplementary material for: Signalling need for care: a neglected functional role of medical treatment
Source: Evol Med Public Health. 2023 Aug 14;11(1):363–78. doi: 10.1093/emph/eoad024 (PMC10611421; doi:10.1093/emph/eoad024)
Supplement: eoad024_suppl_Supplementary_File [file eoad024_suppl_supplementary_file.docx]

# Supplementary File: Undergoing medical treatment legitimises people’s symptoms and sick role: An experimental study on medical overconsumption as a costly signal

## A. Caregiving index

### Items

- This co-worker is definitely ill
- This co-worker is fully committed to getting better
- I will give lots of care and attention to this co-worker until they recover
- My team and I should take over all their work-related responsibilities until they recover fully
- This co-worker deserves to be completely free from their responsibilities for the next week
- It was fully acceptable for them to be late / absent
- It is fair that I take over my co-worker’s duties

### Response cue

“How much do you agree with the following statements?”

### Response options

- Disagree strongly
- Disagree
- Neither agree nor disagree
- Agree
- Agree strongly

## B. Validation of dependent variable used in studies 1 and 2

### Introduction

The caregiving index is a new measure developed for this study. Here we describe a cross-sectional survey to assess the relationship between scores on the caregiving index and other related constructs. These induced a measure people’s emotional response to people in need (The Interpersonal Reactivity Index, empathic concern sub-scale (DAVIS, 1980) a measure peoples belief that helping people is an ethical obligation (The Principle of Care, Wilhelm & Bekkers (2010)), a measure of distrust or cynicism in others (New Machiavellianism Scale, Dahling et al. (2009), distrust of others sub-scale, Gu et al. (2017)) and a measure actual pro-social behaviour (the dictator game / donations to charity Kahneman et al. (1986)).

### Methods

#### Sample

We sought to collect data from 75 people using Prolific, an online participant recruitment platform. As in the main study, recruitment was limited to participants over 25 years old.

#### Measures

##### Caregiving index

The caregiving index scores were measured by presenting participants with a vignette and asking about agreement with a set of items The vignette (condition B / Flu, manipulation level: treatment) was as follows:

Someone that you work with came into work this week sniffling a little. She also has stomach pains and nausea. For various reasons your colleague keeps missing work since she was hired two months ago. It seems she does not enjoy her job very much. And now this week you see she is leaving work early and missing important meetings. Today she has emailed in sick from work and attached a *sick note* from the local doctor. The doctor wrote that her problem was not very serious and that he prescribed a course of strong antibiotics to take for her illness. Once again, you and your colleagues are going to have to work on Saturday to complete her tasks.

The following items were then presented a random order. These items were derived from developed as measures of one’s perceived legitimacy of the target illness and their occupation of the sick role.

- This co-worker is definitely ill
- This co-worker is fully committed to getting better
- I will give lots of care and attention to this co-worker until they recover.
- My team and I should take over all their work-related responsibilities until they recover fully
- This co-worker deserves to be completely free from their responsibilities for the next week
- It was fully acceptable for them to be late / absent
- It is fair that I take over my co-worker’s duties

Participants registered agreement by clicking on boxes labelled disagree strongly; disagree; neither agree nor disagree; agree; or agree strongly.

##### Interpersonal Reactivity Index (empathic concern sub-scale)

Examples items are

- Sometimes I don’t feel very sorry for other people when they are having problems. (reverse coded)
- When I see someone being taken advantage of, I feel kind of protective towards them.

The sub-scale includes seven items and responses were measured on a 1 to 5 scale anchored by “describes me very well” and “Does not describe me well”.

##### Principle of care

This measure is defined as the “moral position that one should help those in need” (Wilhelm & Bekkers, 2010) and has three items measured on with the same agreement responses as the caregiving index:

- People should be willing to help others who are less fortunate.
- Personally assisting people in trouble is very important to me.
- These days people need to look after themselves and not overly worry about others. (reverse coded.)

##### New Machiavellianism Scale (Distrust of others sub-scale)

This scale is “conceptualised as one’s propensity to distrust others, engage in amoral manipulation, seek control over others, and seek status for oneself” (Dahling et al., 2009). Here we examine if the distrust sub-scale predicts lower caregiving index scores. Example items are:

- People are only motivated by personal gain
- I dislike committing to groups because I don’t trust others
- Team members backstab each other all the time to get ahead*

Agreement was measured on with the same response scale as the caregiving index.

##### Donation to charity

To assess the relationship between caregiving index scores and the actual donation behaviour, participants were given the opportunity to donate some, all, or none of this to the Against Malaria Foundation. The text read as follows:

You have finished - well done! We offer a bonus of 50p to all participants who make it this far. You can keep all of the bonus, or give some/all to the Against Malaria Foundation. How much would you like to **keep**? It will be credited to your prolific account.

This measure is equivalent to a dictator game (Kahneman et al., 1986).

#### Procedure

Participants, recruited from Prolific, read the information sheet and consent form and then completing basic demographic questions and the Caregiving Index. They then completed the Interpersonal Reactivity Index, the Principle of Care and the New Machiavellianism Scale (Distrust of others sub-scale) in a random order. Finally, the completed the bonus donation / dictator game.

### Results

76 people (42 women) participated in the study and the mean age was 31.93 (SD = 10.49). Figure S1 shows the relationship between the caregiving index and the other constructs measured.


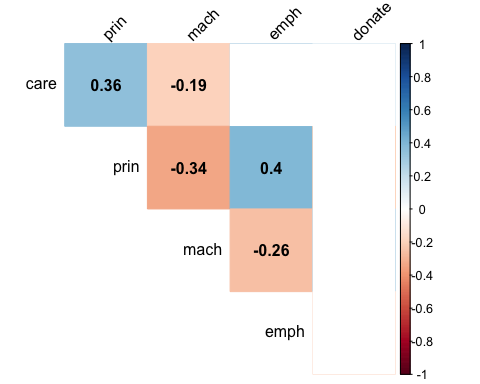


Figure S1: Relationship between caregiving index (care), the Principle of Care (prin), New Machiavellianism Scale, distrust of others subscale (mach), Interpersonal Reactivity Index, empathhic concern subscale (emph), and donations to charity (donate). Correlations in which p-value is less than .1 are coloured.

### Discussion

The caregiving index is associated with principle of care, a measure of people’s ethical position that one should care for people in need. There was also a weak negative relationship with Machiavellianism distrust.

## C. Variables Used in Study 3: exact wording and psychometric properties

The following measures were included:

### A legitimacy index

- There are people in my life who would help me more if they believed in my pain/illness.
- My colleagues at work have a good understanding of my pain/illness.
- If people took my pain/illness more seriously, I would have greater support.
- Other people sometimes think I am lazy rather than sick.
- Some friends and family members are skeptical of my pain/illness.
- People I depend on don’t believe I am committed to recovering from my pain/illness.
- Some doctors/nurses I have interacted with don’t believe I am as ill as I say.

### The SSCI-8, slightly modified:

- Because of my pain/illness, some people seemed uncomfortable with me.
- Because of my pain/illness, some people avoided me.
- Because of my pain/illness, I felt left out of things.
- Because of my pain/illness, people were unkind to me.
- Because of my pain/illness, people avoided looking at me.
- I felt embarrassed about my pain/illness.
- I felt embarrassed because of my physical limitations.
- Some people acted as though it was my fault I have this pain/illness.

### A measure of treatment acceptance.

The first four items assess preferences for benign treatments with low signaling value, the last four items assess preferences for more aversive treatments which presumably have greater signalling value. The item order presentation was randomized between participants.

- I would… undergo weekly therapy sessions with a therapist.
- I would… do 1 hour of intense exercise every single day.
- I would… download and use a pain-related smartphone app for 30 minutes every day.
- I would… read and implement the lessons in a self-help book for half an hour per day.
- I would… undergo a minor surgery which will result in some visible scarring.
- I would… take a new but potentially dangerous kind of painkiller medication.
- I would… have a surgical implant for pain reduction.
- I would… take a drug that carries a risk of stroke.

### Scale psychometrics for Study 3

| Variable | alpha | theoretical range | range | mean | SD | median |
| --- | --- | --- | --- | --- | --- | --- |
| Legitimacy (3a) | 0.81 | 1 to 7 | 1 to 7 | 3.7 | 1.5 | 3.8 |
| Stigma (SSCI-8) (3a) | 0.86 | 1 to 5 | 1 to 4.8 | 2.2 | 0.8 | 2.0 |
| Benign treatment acceptance (3a) | 0.65 | 1 to 7 | 1 to 7 | 4.3 | 1.5 | 4.1 |
| Aversive treatment acceptance (3a) | 0.74 | 1 to 7 | 1 to 7 | 2.8 | 1.5 | 2.5 |
| Benign treatment acceptance (3b) | 0.72 | 1 to 7 | 1 to 7 | 4.9 | 1.4 | 5.0 |
| Aversive treatment acceptance (3b) | 0.74 | 1 to 7 | 1 to 7 | 2.8 | 1.5 | 2.5 |
| Benign treatment acceptance (3c) | 0.73 | 1 to 7 | 1 to 7 | 4.6 | 1.4 | 4.8 |
| Aversive treatment acceptance (3c) | 0.74 | 1 to 7 | 1 to 7 | 2.5 | 1.4 | 2.5 |

Note one item excluded from the legitimacy measure in 3a is participants did not appear to notice the reverse coding. Removing the item increased the alpha from .78 to .81 and the average correlation between this item and the other items was .36 (>=.63 for all other items). Results are similar with or without the item.

## D. Preregistered analysis of Study 3a

A reviewer pointed to a more straightforward approach to the slightly unworldly approach specified in the preregistration. The qualitative results are similar and we present the preregistered analyses here:

Testing the main hypothesis, a multiple regression model confirms that high stigma and low legitimacy predict a preference for medical treatments, particularly aversive treatments (see Tables 2 and 3).

|  | **Aversive treatments** | | | **Benign treatments** | | |
| --- | --- | --- | --- | --- | --- | --- |
| *Coeffcient* | *Estimates* | *CI* | *p* | *Estimates* | *CI* | *p* |
| Intercept | 0.36 | -0.61 – 1.32 | 0.465 | 3.83 | 2.79 – 4.87 | **<0.001** |
| Stigma | 0.53 | 0.26 – 0.80 | **<0.001** | 0.30 | 0.01 – 0.59 | **0.043** |
| Pain (intensity) | 0.01 | 0.00 – 0.02 | **0.038** | -0.01 | -0.02 – 0.00 | 0.125 |
| Pain (disability) | 0.01 | -0.00 – 0.02 | 0.121 | 0.01 | 0.00 – 0.02 | **0.015** |
| Observations | 182 | | | 182 | | |
| R^2^ / R^2^ adjusted | 0.189 / 0.175 | | | 0.092 / 0.077 | | |

Table 2 Does stigma predict treatment use preferences?

|  | **Aversive treatments** | | | **Benign treatments** | | |
| --- | --- | --- | --- | --- | --- | --- |
| *Coeffcient* | *Estimates* | *CI* | *p* | *Estimates* | *CI* | *p* |
| Intercept | 0.54 | -0.39 – 1.47 | 0.252 | 4.00 | 2.99 – 5.01 | **<0.001** |
| Illness illegitmacy | 0.27 | 0.13 – 0.40 | **<0.001** | 0.12 | -0.03 – 0.27 | 0.112 |
| Pain (intensity) | 0.01 | -0.00 – 0.02 | 0.156 | -0.01 | -0.02 – 0.00 | 0.076 |
| Pain (disability) | 0.01 | 0.00 – 0.02 | **0.003** | 0.02 | 0.01 – 0.03 | **0.001** |
| Observations | 182 | | | 182 | | |
| R^2^ / R^2^ adjusted | 0.189 / 0.175 | | | 0.084 / 0.069 | | |

Table 3 Does legitimacy predict treatment use preferences?

Signalling theory predicts that stigma/legitimacy should explain more variance in aversive treatment use in than benign treatment use since the benign treatments are less able to signal authenticity. Using legitimacy and stigma as the dependent variables and benign/aversive treatment use as the predictors variables, we can use AICs to compare regression models and test this prediction. As predicted, a preference for aversive treatments provided a better fit for illness legitimacy (AIC difference = 20) and for stigma (AIC difference = 21) than a preference for benign treatments.

# References

Dahling, J. J., Whitaker, B. G., & Levy, P. E. (2009). The development and validation of a new machiavellianism scale. *Journal of Management*, *35*(2), 219–257.

DAVIS, M. (1980). A muntidimensional approach to individual differences in empathy. *JSAS Catalogue of Selected Documents in Psychology*, *10*.

Gu, H., Wen, Z., & Fan, X. (2017). Structural validity of the machiavellian personality scale: A bifactor exploratory structural equation modeling approach. *Personality and Individual Differences*, *105*, 116–123.

Kahneman, D., Knetsch, J. L., & Thaler, R. H. (1986). Fairness and the assumptions of economics. *Journal of Business*, S285–S300.

Wilhelm, M. O., & Bekkers, R. (2010). Helping behavior, dispositional empathic concern, and the principle of care. *Social Psychology Quarterly*, *73*(1), 11–32.
